# Intermittent Recurrence of Ureterosciatic Hernia After Spontaneous Resolution, Complicated by Emphysematous Pyelonephritis

**DOI:** 10.1002/iju5.70096

**Published:** 2025-09-14

**Authors:** Akira Ohtsu, Keisuke Hori, Seiji Arai, Yuki Morimura, Ayaka Igarashi, Yuji Fujizuka, Yoshitaka Sekine, Hidekazu Koike, Hiroshi Matsui, Kazuhiro Suzuki

**Affiliations:** ^1^ Department of Urology Gunma University Hospital Maebashi Gunma Japan

**Keywords:** emphysematous pyelonephritis, hydronephrosis, ureteral realignment, ureteral stent, ureterosciatic hernia

## Abstract

**Introduction:**

Ureterosciatic hernia is a rare condition in which the ureter herniates through the sciatic foramen, causing ureteral obstruction, and urinary infection.

**Case Presentation:**

A 72‐year‐old woman presented with left flank pain. Computed tomography revealed left hydronephrosis with ureterosciatic hernia. During treatment planning, she developed a cerebral infarction and worsening angina, and received conservative management. One year later, computed tomography showed spontaneous resolution of the hernia. Subsequently, she experienced left flank pain, and repeat computed tomography demonstrated recurrence of the hernia with emphysematous pyelonephritis. Due to the high surgical risk, she underwent antibiotic therapy and stepwise retrograde ureteral realignment with ureteral catheter insertion, followed by double‐J stent replacement.

**Conclusion:**

This is the first report to describe the spontaneous resolution and intermittent recurrence of ureterosciatic hernia on serial radiographic examinations. Retrograde ureteral realignment may be a safe and effective alternative to surgical repair in frail patients.

**Summary**

- Ureterosciatic hernia exhibits a dynamic clinical course. Ureteral stenting is an effective treatment for ureterosciatic hernia.
- For frail patients in whom definitive surgical repair is contraindicated, retrograde ureteral stenting offers a viable alternative.

## 1 Introduction

Ureterosciatic hernia (USH) is a rare condition in which the ureter herniates through the greater or lesser sciatic foramen, resulting in ureteral obstruction and, in some cases, severe urinary infection. Approximately 50 cases have been documented so far, representing a small fraction of the underlying diseases for ureteral obstructions worldwide [1, 2]. The clinical presentation can range from asymptomatic hydronephrosis to life‐threatening urosepsis, while the physical findings are generally nonspecific. Although cross‐sectional imaging, particularly contrast‐enhanced computed tomography (CT), plays a central role in radiological diagnosis, the dynamic and sometimes intermittent nature can lead to misinterpretations or delays in clinical recognition. Here, we report a rare case of left USH in an older woman with significant comorbidities. The case is unique because it captures the spontaneous resolution and intermittent recurrence of USH, accompanied by severe infection, observed on serial CT examinations.

## 2 Case Presentation

**FIGURE 1:**
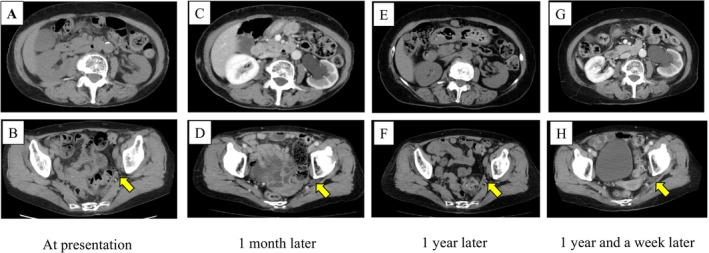
Spontaneous resolution and intermittent recurrence of USH. (A–H) CT images at four different time points: At presentation (A, B), 1 month later (C, D), 1 year later (E, F), and 1 year and 1 week later (G, H). The upper panels show axial images at the kidney level, and the lower panels show axial images at the pelvic level. The yellow arrows indicate the location of the left ureter.

**FIGURE 2:**
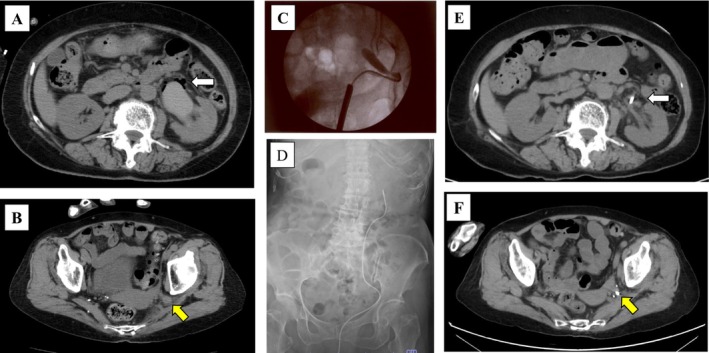
Radiographic images during the perioperative period for ureteral stenting. (A, B) Axial CT images of the emphysematous changes in the left pelvis (white arrow) and left ureter (yellow arrow). (C) Intraoperative fluoroscopic image of the left USH. (D) Plain abdominal radiograph after left ureteral catheter insertion. (E, F) Axial CT images at 4 days after ureteral catheter insertion. White arrow indicates left pelvis, and yellow arrow indicates left ureter.

**FIGURE 3:**
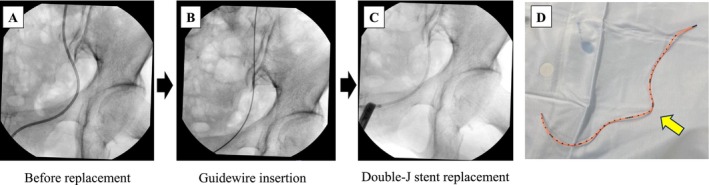
Ureteral double J‐stent replacement procedure. (A–C) Fluoroscopic images showing sequential steps: Ureteral catheter before replacement (A), successful relocation of the left ureter with guidewire insertion (B), and final double‐J stent replacement in the left ureter (C). (D) Image of the removed ureteral catheter. The yellow arrow indicates the corresponding site of the hernia orifice.

**FIGURE 4:**
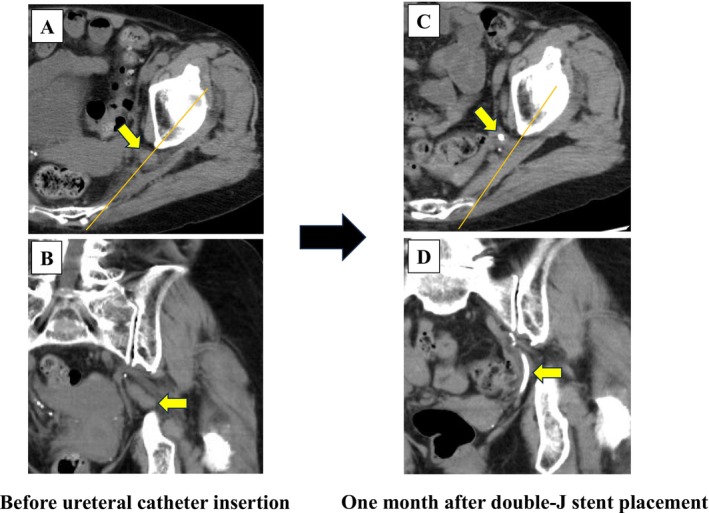
Serial CT images before and after resolution of USH. (A, B) CT images of left USH before ureteral catheter placement (top: Axial view; bottom: Coronal view). (C, D) CT images at 1 month after double‐J stent replacement (top: Axial view; bottom: Coronal view). The orange lines indicate the line connecting the ischium and sacrum. The yellow arrows indicate the left ureter.

A 72‐year‐old woman with a history of angina pectoris presented to her primary care physician with a 6‐month history of intermittent left flank pain. CT showed no abnormalities in the left kidney (Figure 1A,B). However, her symptoms remained, and contrast‐enhanced CT performed 1 month later revealed left hydronephrosis (Figure 1C,D). Thus, she was referred to our institution for further evaluation. At the time of presentation, her clinical symptoms were not severe, and a review of the sequential CT examinations indicated left USH as the cause of the hydronephrosis. [^99m^Tc]‐mercaptoacetyltriglycine renal scintigraphy confirmed preserved function of the left kidney (Figure S1). While treatment options were considered, she developed a cerebral infarction. Thereafter, she underwent coronary artery bypass surgery for worsening angina and subsequently developed an abdominal wall hernia at the caudal side of the surgical wound. Given her multiple comorbidities and mild symptoms, conservative management with close follow‐up was selected. Approximately 1 year later, follow‐up CT unexpectedly showed no hydronephrosis in the left kidney, indicating spontaneous resolution of USH (Figure 1E,F). However, 1 week later, she visited an affiliated hospital with vomiting and left flank pain, and CT revealed recurrence of left hydronephrosis (Figure 1G,H). Laboratory data showed no evidence of infection, and she received conservative treatment. Four days later, she presented to our hospital with fever and general malaise. Laboratory data revealed a normal white blood cell count (8500/μL) but elevated C‐reactive protein (27.73 mg/dL). CT confirmed recurrence of left USH with emphysematous pyelonephritis (Figure 2A,B). Due to ongoing antiplatelet therapy for the cerebral infarction and angina pectoris, we opted for retrograde ureteral stenting using a cystoscope rather than percutaneous nephrostomy. Ureterography showed a characteristic “curlicue” ureter sign (Figure 2C). A 6‐Fr open‐ended ureteral catheter was successfully advanced into the renal pelvis, allowing direct renal pelvic irrigation as needed and facilitating effective drainage of pyuric discharge (Figure 2D). At this time, intravenous broad‐spectrum antibiotic treatment using piperacillin‐tazobactam (2.25 g every 8 h for 4 days) was initiated and subsequently de‐escalated with ceftriaxone (2 g/day for 9 days), based on the urinary culture results ( *Escherichia coli* ). Four days later, CT indicated marked improvement in the hydronephrosis and emphysematous changes (Figure 2E,F). At 2 weeks after ureteral drainage and antibiotic treatment, the ureteral catheter was replaced with a 6‐Fr, 24‐cm double‐J stent (Figure 3A–D). The removed catheter was sharply kinked at the herniation site (Figure 3E), corroborating ureteral compression. Comparisons of the CT images before and after ureteral stenting showed relocation of the left ureter and resolution of USH (Figure 4A–D). Although ureteral stent removal or definitive surgical repair could be considered after the resolution of USH, the patient continues to undergo scheduled ureteral stent exchanges every 3 months because of the risk of recurrent emphysematous pyelonephritis and significant comorbidities. No recurrence of hernia or severe infection has been observed in > 24 months of follow‐up. Moreover, the renal function remained stable over time (Figure S2).

## 3 Discussion

USH is a rare and under‐recognized cause of ureteral obstruction, primarily affecting the left ureter in elderly and thin women. This may be due to the longer left ureter length, wider pelvic anatomy, and increased abdominal pressure caused by constipation [2, 3, 4, 5]. Specifically, age‐related atrophy of the piriformis and obturator internus muscles, along with weakening of the pelvic fascial support, could facilitate ureteral herniation through the greater sciatic foramen [5]. The present case fits these demographic and anatomical characteristics. To our knowledge, this is the first case to document the spontaneous resolution and intermittent recurrence of USH with emphysematous pyelonephritis, observed on serial CT examinations.

Accurate diagnosis of USH relies on cross‐sectional imaging. CT remains the gold standard, particularly in the presence of complications such as hydronephrosis or emphysematous changes [6]. Three‐dimensional reconstructions can be helpful for preoperative planning. MR urography could have been a radiation‐free alternative for follow‐up, and ultrasonography could have facilitated indirect surveillance by tracking the hydronephrosis [5].

Conservative management for USH can be appropriate for asymptomatic patients with preserved renal function [7]. Nevertheless, close monitoring is necessary, because delayed severe infections sometimes occur. Indeed, functional assessment by MAG3 renography in our patient confirmed the absence of significant obstruction, thereby supporting an initial conservative approach [8]. The subsequent development of emphysematous pyelonephritis highlights the potential for delayed but serious complications, even in mild symptomatic cases. This underscores the importance of close imaging follow‐up and timely intervention in patients with nonsurgical management.

Surgical management strategies for USH, including retrograde ureteral stenting, percutaneous nephrostomy with antegrade ureteral stenting, and definitive surgical repair, are essential for patients with severe symptoms or an infection [5, 9, 10, 11, 12]. While retrograde ureteral stenting is a minimally invasive option, USH can sometimes recur [9], as observed in the present case. Meanwhile, surgical repair with mesh hernioplasty provides a permanent solution but requires general anesthesia, which is not always feasible in patients with severe comorbidities [10, 11, 12]. The present patient had a high Charlson Comorbidity Index of 6 with abdominal wall hernia and was on antiplatelet therapy, rendering her a poor candidate for definitive surgery. We adopted a stepwise retrograde approach, comprising initial guidewire‐assisted insertion of a ureteral stent, followed by double‐J stent replacement, to avoid the morbidity associated with definitive surgery. Moreover, the initial catheter placement allowed direct renal pelvis irrigation, which likely facilitated the clearance of viscous pyuria, contributing to the resolution of the emphysematous changes.

In conclusion, this is the first case to document the spontaneous resolution and infectious recurrence of USH using serial CT examinations. A stepwise retrograde approach provided prompt infection control and durable ureteral realignment without the need for definitive surgery. This strategy may serve as a practical and effective alternative to surgical repair in patients with infection‐associated USH who are poor surgical candidates.

## Consent

Written informed consent was obtained from the patient to publish this case report and any accompanying images. Proof of consent to publish from the patient can be requested at any time.

## Conflicts of Interest

The authors declare no conflicts of interest.

## Supporting information


**Figure S1:** MAG3 renogram showing no functional difference or delayed voiding pattern in the bilateral kidneys.
**Figure S2:** Time course of estimated glomerular filtration rate (eGFR) following the patient's initial visit.
