# Supplementary material for: Intermittent Recurrence of Ureterosciatic Hernia After Spontaneous Resolution, Complicated by Emphysematous Pyelonephritis
Source: IJU Case Rep. 2025 Sep 14;8(6):591–5. doi: 10.1002/iju5.70096 (PMC12580005; doi:10.1002/iju5.70096)
Supplement: Supplementary file 1 — Figure S1: MAG3 renogram showing no functional difference or delayed voiding pattern in the bilateral kidneys. Figure S2: Time course of estimated glomerular filtration rate (eGFR) following the patient's initial visit. [file IJU5-8-591-s001.pptx]

## Slide 1
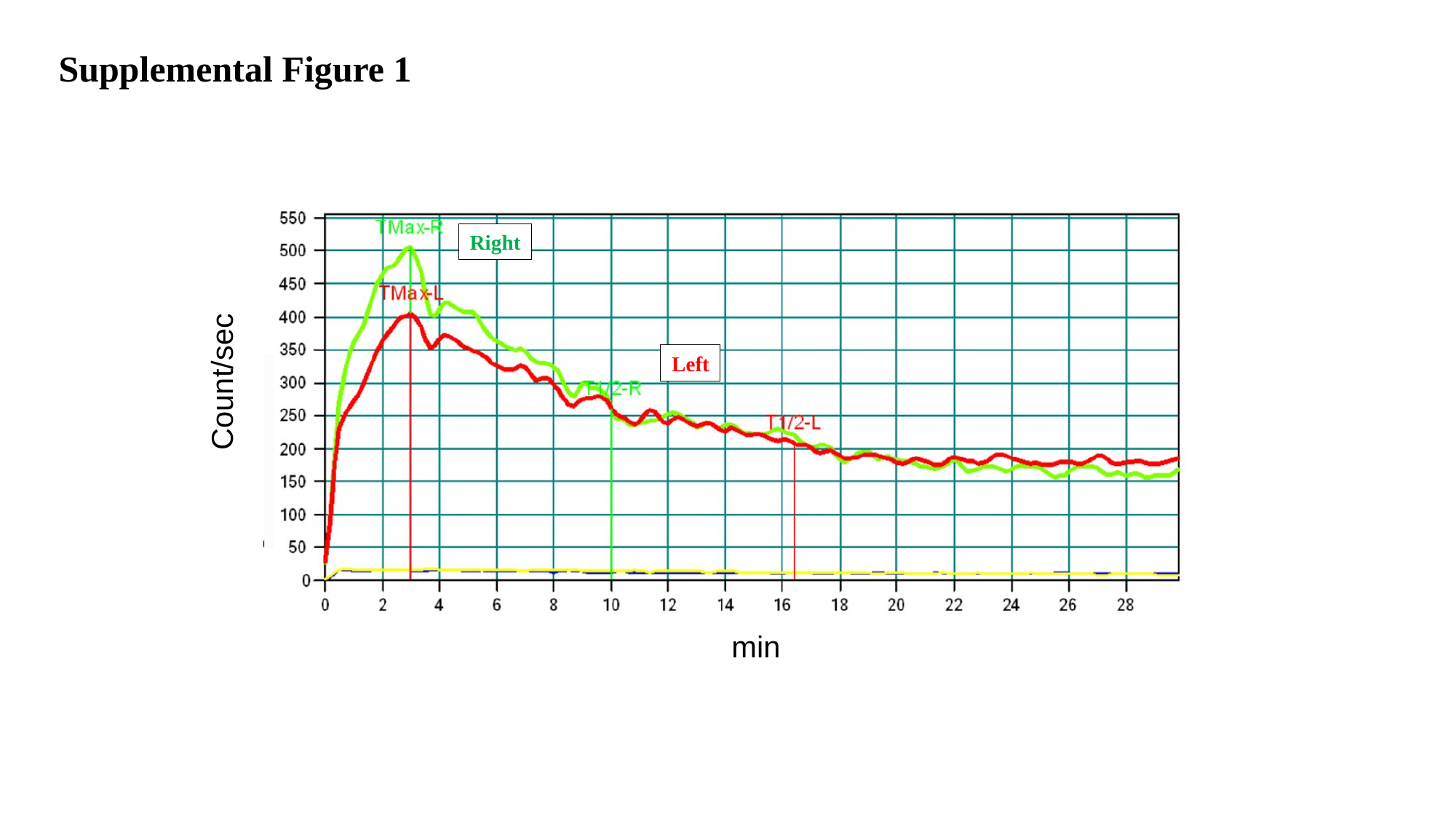

Supplemental Figure 1
Right
Left
Count/sec
min

## Slide 2
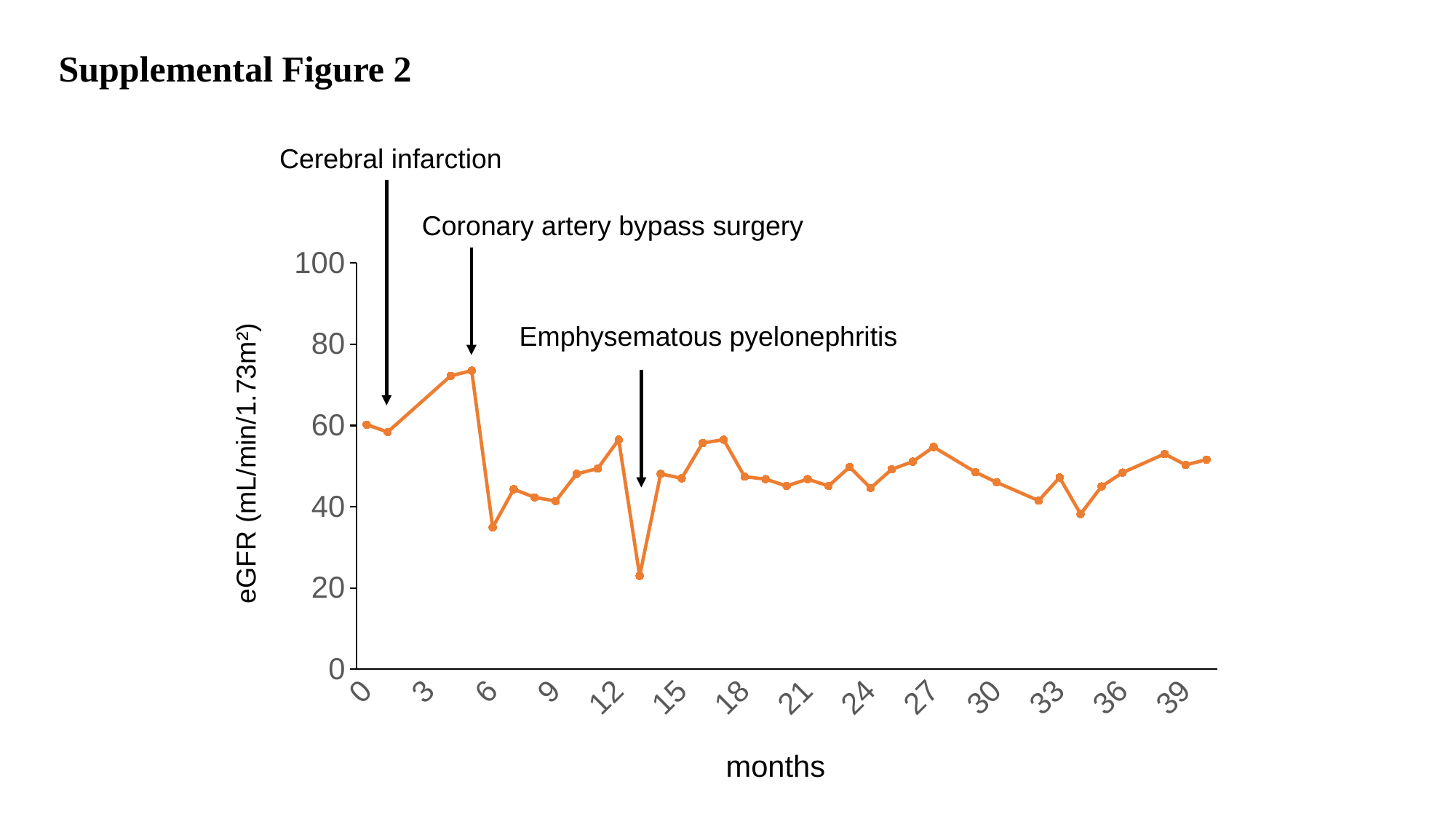

Supplemental Figure 2
Cerebral infarction
### Chart
| Category | eGFR |
|---|---|
| 0 | 60.2 |
| 1 | 58.4 |
| 2 | None |
| 3 | None |
| 4 | 72.2 |
| 5 | 73.5 |
| 6 | 34.9 |
| 7 | 44.3 |
| 8 | 42.3 |
| 9 | 41.4 |
| 10 | 48.1 |
| 11 | 49.4 |
| 12 | 56.5 |
| 13 | 23.0 |
| 14 | 48.1 |
| 15 | 47.0 |
| 16 | 55.7 |
| 17 | 56.5 |
| 18 | 47.4 |
| 19 | 46.8 |
| 20 | 45.1 |
| 21 | 46.8 |
| 22 | 45.1 |
| 23 | 49.8 |
| 24 | 44.6 |
| 25 | 49.2 |
| 26 | 51.1 |
| 27 | 54.7 |
| 28 | None |
| 29 | 48.5 |
| 30 | 46.0 |
| 31 | None |
| 32 | 41.5 |
| 33 | 47.2 |
| 34 | 38.2 |
| 35 | 45.0 |
| 36 | 48.4 |
| 37 | None |
| 38 | 53.0 |
| 39 | 50.3 |
| 40 | 51.6 |Coronary artery bypass surgery
Emphysematous pyelonephritis
eGFR (mL/min/1.73m²)
months
